# COVID-19 Presenting As Miller Fisher Syndrome in a Patient With a History of Guillain-Barré Syndrome: A Case Report

**DOI:** 10.7759/cureus.26588

**Published:** 2022-07-05

**Authors:** Louay Aldabain, Metri Haddaden, Rehan Farooqi, Malaz Alissa

**Affiliations:** 1 Hospital Medicine, MedStar Good Samaritan Hospital, Baltimore, USA; 2 Internal Medicine, MedStar Union Memorial Hospital, Baltimore, USA

**Keywords:** anti gq1b antibodies, ophthalmoplegia, ataxia, diplopia, miller fisher syndrome, covid-19

## Abstract

Coronavirus disease 2019, caused by severe acute respiratory syndrome coronavirus 2, primarily affects the respiratory system. While coronaviruses are not a common cause of neurological disease, they have been reported to cause direct central nervous system infection, as well as presumed para-infectious disorders. Here we report a very rare case of SARS-CoV-2 infection presenting as Miller Fisher syndrome with positive anti-GQ1b antibodies in a patient with a history of Guillain-Barré syndrome, which was treated with IV immunoglobulin resulting in marked improvement in her symptoms. Thus, a high index of suspicion and meticulous observation are the cornerstones to identifying possible uncommon presentations of COVID-19.

## Introduction

Severe acute respiratory syndrome coronavirus 2 (SARS-CoV-2), the causative agent of coronavirus disease 19 (COVID-19), primarily infects the respiratory system and results in a spectrum of presentations ranging from being entirely asymptomatic to acute respiratory distress syndrome (ARDS) [1]. In addition, there has been growing evidence of non-respiratory complications related to COVID-19, including gastroenterological, hematological, nephrological, and neuropsychiatric symptoms [2]. While the pathophysiology explaining the former manifestations remains unclear, it is thought to be the result of an interplay between the virus and the host immune system [3].

Although it is not uncommon for COVID 19 to present with neurological manifestations, Miller Fisher syndrome (MFS), a rare variant of Guillain-Barré syndrome (GBS), secondary to COVID-19, has only been reported a handful of times in the literature [4-15]. Here we report a unique case of coronavirus disease 19 presenting as Miller Fisher syndrome with positive anti-GQ1b antibodies in a patient with a history of Guillain-Barré syndrome.

## Case presentation

A 39-year-old woman with a past medical history of hypertension, diabetes mellites type 2, and GBS presented to the emergency department with diplopia, progressive bilateral upper and lower extremity weakness and numbness, and ataxia of two-week duration. She denied any recent infection or receiving any vaccinations. On physical examination, her blood pressure was 173/83 mmHg, and heart rate was 74 beats per minute. She was afebrile and saturating 100% on room air. She had left eye medial conjunctival erythema, deconjugate gaze, inability to abduct her left eye, inability to abduct her right eye with minimal adduction, elevation, and depression. She was unable to puff her cheeks bilaterally. Her strength was 4/5 in the proximal upper and lower extremities, length-dependent decrease in temperature sensation in the lower extremity starting from the knee bilaterally. Her deep tendon reflexes were +1 in triceps bilaterally, 0 in patella and ankles bilaterally, and a negative Babinski sign.

Pertinent laboratory tests at that time showed unremarkable complete blood count (CBC), complete metabolic panel (CMP), urine analysis (UA), thyroid-stimulating hormone (TSH), beta-human chorionic gonadotropin (BhCG), creatinine kinase (CK), serum protein electrophoresis (SPEP), human immunodeficiency virus 1/2 antibody/antigen (HIV AB/AG), syphilis screen, and vitamins B1, B6, and B12. Mid-turbinate COVID-19 direct real-time reverse transcription-polymerase complete reaction (RT-PCR) assay was positive. Table 1 shows antibody work-up.

**Table 1:** Antibody work-up

Antibody	Result
Sox 1 antibody IgG immunoblot.	Negative
CV 2.1 antibody IgG screen	Negative
Purkinje/neuronal nuclear IgG screen	Negative
neuronal antibody (amphiphysin)	Negative
Asialo -GM1B antibody IgG/IgM	Negative
GM1 antibody IgG/IgM	Negative
GM2 antibody IgG/IgM	Negative
GD1a antibody IgG/IgM	Negative
GD1B antibody IgG/IgM	Negative
GQ1B antibody IgG/IgM	397 mg/dL
IgG	1488 mg/dL

Magnetic resonance imaging (MRI) of the brain and spine with and without contrast, brain magnetic resonance angiography (MRA), and brain magnetic resonance venography (MRV) were all normal. A nerve conduction study showed electrophysiological evidence of a right median sensory neuropathy. Electromyography (EMG) showed evidence of reduced recruitment in the left tibialis anterior as well as the left vastus lateralis (Table 2). Cerebrospinal fluid (CSF) analysis, including cultures, oligoclonal bands, immunoglobulin G (IgG) index, cytology, and flow cytometry, were unremarkable, with no protein-albumin dissociation.

**Table 2:** Electromyogram (EMG) EMG: Electromyogram, MUAP: motor unit action potential, IA: insertion activity, Fib: fibrillation, PSW: polyspike wave, Fasc: fasciculations, H.F: high frequency, Amp: amplitude, Dur: duration, PPP: polyphasic potentials, N: normal, L left, R: right, Tib: tibialis, Vast: Vastus, Abd: abductor, Poll: Pollicis

	Spontaneous					MUAP			Recruitment
	IA	Fib	PSW	Fasc	H.F.	Amp	Dur	PPP	Pattern
L tib anterior	N	None	None	None	None	N	N	N	Reduced
L vast lateralis	N	None	None	None	None	N	N	N	Reduced
R deltoid	N	None	None	None	None	N	N	N	N
R biceps	N	None	None	None	None	N	N	N	N
R abd poll brevis	N	None	None	None	None				
L deltoid	N	None	None	None	None	N	N	N	N

The patient was diagnosed with MFS in the setting of COVID-19 and was started on intravenous immunoglobulins (IVIG) at a dose of 20 g/day for five days, along with alternating patching of each eye. After treatment completion, she had subjective and objective improvement in her symptoms, and she was eventually discharged to an acute rehabilitation facility.

## Discussion

MFS is an autoimmune disease characterized by a triad of ophthalmoplegia, ataxia, and areflexia. It is typically associated with high serum anti-GQ1b IgG in up to 83% of cases [16]. It accounts for 1-5% of cases of GBS in Western countries and is usually precipitated by preceding infections such as *Campylobacter jejuni*, and *Haemophilus influenzae* due to antigenic mimicry between peripheral nerves and the inciting microbe [17,18].

The case we describe is an unusual initial presentation of COVID-19 manifesting as MFS. The writers of this article did a Medline and PubMed Search and found that there are only twelve other cases of COVID-19-induced MFS [4-15]. We compared the findings in our study with the other studies for presentation, antibodies, MRI findings, and recurrence. Our case is the second case in the United States and the third case worldwide to report MFS as the initial presentation in a patient infected with COVID-19 [7,14]. All other cases reported COVID-19 symptoms preceding MFS presentation [4-6,8-13,15]. Including our case, only four cases of COVID-19-induced MFS reported positive anti-GQ1b antibodies [5,13,14]. The GQ1b ganglioside is a cell surface component concentrated in the paranodal regions of the extramedullary portion of CN III, IV, and VI. Antibodies against this ganglioside are thought to be the cause of ophthalmoplegia in MFS [19].

Brain MRI is not required for MFS diagnosis since findings are usually normal, and abnormalities are only detected in 2% of patients [20]. Three cases of COVID-19-induced MFS reported MRI findings which included prominent enhancement with gadolinium and T2 hyperintense signal of the cranial nerve III [6], enhancement, T2-hyperintensity, and enlargement of the left oculomotor nerve [4], and intrathe­cal cauda-equina enhancement [5].

Lastly, it is essential to note that among all the reported cases, only our patient had a remote history of GBS before presenting with MFS- induced COVID 19. It is unclear if such a history increases the likelihood of MFS as a complication of COVID-19. Further studies are required to confirm the former.

## Conclusions

We continue to learn more about the unexpected course the new virus might follow. Thus, a high index of suspicion and meticulous observation are the cornerstones to identifying possible uncommon presentations. Limited data is available in the literature on the association between Miller fisher syndrome and coronavirus disease 2019. Therefore, further research is required to enhance our understanding of the pathophysiology of such association. With that being said, we believe that this case provides a crucial addition to the collective knowledge about both the disease and its complications.
